# Correction: A case control study to investigate differences in motor control between individuals with and without non-specific low back pain during standing

**DOI:** 10.1371/journal.pone.0270017

**Published:** 2022-06-09

**Authors:** Cathrin Koch, Augusto Garcia-Agundez, Stefan Göbel, Frank Hänsel

The second author’s name is spelled incorrectly. The correct name is: Augusto Garcia-Agundez. The correct citation is: Koch C, Garcia-Agundez A, Göbel S, Hänsel F (2020) A case control study to investigate differences in motor control between individuals with and without non-specific low back pain during standing. PLoS ONE 15(7): e0234858. https://doi.org/10.1371/journal.pone.0234858
