# Supplementary material for: A case control study to investigate differences in motor control between individuals with and without non-specific low back pain during standing
Source: PLoS One. 2020 Jul 6;15(7):e0234858. doi: 10.1371/journal.pone.0234858 (PMC7337307; doi:10.1371/journal.pone.0234858)
Supplement: Attachment — Submitted filename: Response to Reviewers_Koch et al..docx [file pone.0234858.s001.docx]

Reviewer #1: I really appreciate the study for its Innovative correlation pattern. I would like to recommend using the visual analogue scale analysis in the future similar trials so that a quantitative estimation of the NSLBP can enrich the correlation.

Response: Thank you for your suggestion. We have considered the use of the VAS. To avoid overburdening participants we decided to use the numeric rating scale. Nonetheless your argument for the VAS is convincing. For the future, we plan on using the VAS to enrich the correlation.

Reviewer #2: This manuscript documents a study comparing those with non-specific low back pain (NSLBP) to age- and sex-matched healthy control participants in terms of trunk muscle activity and centre of pressure control during three quiet standing conditions. The authors were unable to determine any group differences but did show that one of their measures was affected by standing condition despite showing some observable differences in descriptive statistics.

I believe there is scientific merit in this manuscript, but the writing and presentation needs substantial improvement to improve the quality and clarity of this work. The majority of my comments reflect minor technical additions/omissions, however I still suggest a major revision to emphasize the amount of work needed to improve the language.

Response: Thank you for your suggestions on language improvement. The manuscript has been reviewed by a native English speaker.

General Comments
The manuscript is filled with grammatical and phrasing errors that make the manuscript difficult to read and understand. I recommend the authors have a fluent English speaker completely review the grammar and phrasing. Examples of particularly difficult to parse passages include, but are not limited to, lines 54-55, 62-68, 74-76, 84-86, 132, 219-220, 305-309.

Response: The manuscript has been reviewed by a native English speaker.

The authors did a good job documenting their NSLBP group, but it appears to be heterogeneous. Most of the prior work the authors cited in their introduction focused on specific subgroups of the NSLBP population. An advantage to this technique is that those other authors can focus on a specific aspect of low back pain development. The purpose of this papers states that the authors wanted to focus on altered motor control, and their standing conditions appear to reflect that. However, their NSLBP population may have been too generic to truly suss out mechanisms of low back pain development related to altered motor control despite controlling for . Subgroups including the Stabilization/Recurrent group in the Treatment-based Classification, parts of the Movement System Impairment paradigm, or the updated O’Sullivan classification scheme (what the authors cited in the discussion in lines 291-300) are more specific and more likely to show a common deviation with respect to a control group. Can the authors rationalize the lack of subgrouping their NSLBP group?

Response: Prior to this case-control study we conducted a review on motor control in individuals with LBP. In the current study our goal was to replicate those findings with a high quality study. The studies included in our Review classify non-specific back pain according to the duration of pain, pain area, or degree of severity. Based on the common classifications in these studies we defined these criteria precisely to generate a homogenous group.

We think it is a valuable comment to consider subgroups as we have now pointed out in our discussion. A further validation here is necessary, but it would be interesting to incorporate subgroups, like for example O´Sullivan suggests, in our further research.

The use of RMS to examine centre of pressure data is common, however it is more sensitive high-frequency changes than a linear measure such as the average or mean absolute value. The authors did not filter their force plate data and collected the data at a high sample rate relative to the frequency content of their signal (quiet standing). This may have washed out potential true differences between their NSLBP and control groups, with the effects more exaggerated the further participants stood away from the force plate origin. I suggest the authors use a metric less sensitive to high-frequency noise (such as the mean absolute value) or remove the higher frequency content prior to computing the RMS on their centre of pressure data. The authors should also report where participants stood relative to the force plate origin.

Response: Standing position includes that participants were standing in the middle of force plate with their medial malleolus at the center with the same distance for the right and left foot. We have added this detail to our manuscript.

Correctly, you mention the problems that occur with the use of the RMS, but since our goal was to make the results of our study comparable to those of other studies, we decided to use RMS, which is the method in the vast majority of studies.

Additionally, we have now applied a 5Hz low pass filter.

Specific Comments
Line 58 – the differences in trunk muscle activity and centre of pressure findings precede discomfort development in this group

Response: We have made changes here to address this comment.

Line 69 – The paragraph starting here seems unnecessarily long to convey the motivation behind use of centre of pressure or EMG data

Response: We have shortened this paragraph.

Line 128 – p’s ≥ 0.210?

Response: We have added an apostrophe.

Line 137 – Are these tasks too easy to differentiate control and NSLBP groups?

Response: This is a good point that we have considered. We mention it in the discussion now. Nonetheless our aim was to investigate quiet standing as specific subgroup of standing and there are previous studies that have found differences in quiet standing as our review shows.

Line 143 – More detail is required about the mood state questionnaire.

Response: We have specified the questionnaire. It’s only purpose was to document stress during the tests, so that we could be assured that the stress itself of the test was not having any impact on the participants, or results.

Line 144 – What was the maximum rating of the numeric rating scale?

Response: 0-10; we added this into the description.

Line 150 – What were the kinematic data collected? Does this refer to the force plate data? If so, “kinematic” is not required, otherwise, please describe the other kinematic data.

Response: Yes, it is only force plate data. We have changed the term.

Line 175 – How did the authors remove heart rate contamination from their EMG data? It is also unclear when this occurred in the data analysis relative to the subMVC normalization.

Response: We added the concrete bandpass filter.

Line 178 – The statistical modelling description seems out of place in the Results section, consider moving back to the Methods.

Response: We have discussed and understand your point. We decided to remove this sentence completely since it is common practice. We also report the p-values.

Lines 188 to 192 – Which numbers correspond to the control group, and which numbers match the NSLBP group?

Response: We have clarified that in this section.

Line 195 – I believe group should be a between-subjects factor, not a repeated factor

Response: Since we matched the groups we can treat them as paired groups. Therefore, we have kept it as a repeated factor. We now point out more clearly that the groups were matched.

Line 228 – Can the authors report an effect size for the non-significant between-group difference in centre of pressure data?

Response: We added an effect size for the non-significant between group difference in center of pressure.

Line 229 – Why is higher centre of mass sway bad? Were participants instructed to stand as still as possible?

Response: Yes, participants were instructed to stand as still as possible. We added this in the methods section

Line 239 – This concluding sentence requires more information.

Response: We provided more information to make the sentence clearer.

Lines 258 to 268 – This paragraph is seems distracted and does not add much to the overall discussion as is.

Response: We recognize that our argument might not be clear enough. In this paragraph we now introduce the idea that different balance strategies depend on the demand of the task, and emphasize that for low demand tasks, minimal use of resources can lead to non-significant results. From our perspective, this is an important point to better classify our results. Therefore, we have shortened the paragraph and clarified our argument.
